# How decisions and the desire for coherency shape subjective preferences over time

**DOI:** 10.1016/j.cognition.2020.104244

**Published:** 2020-07

**Authors:** Adam N. Hornsby, Bradley C. Love

**Affiliations:** a Dunnhumby, 184 Shepherds Bush Road, London W6 7NL, United Kingdom; b Department of Experimental Psychology, University College London, London WC1H 0AP, United Kingdom; c The Alan Turing Institute, United Kingdom

**Keywords:** Preference learning, Decision making, Intrinsic motivation, Free choice, Choice-induced preference change

## Abstract

Recent findings suggest a bidirectional relationship between preferences and choices such that what is chosen can become preferred. Yet, it is still commonly held that preferences for individual items are maintained, such as caching a separate value estimate for each experienced option. Instead, we propose that all possible choice options and preferences are represented in a shared, continuous, multidimensional space that supports generalization. Decision making is cast as a learning process that seeks to align choices and preferences to maintain coherency. We formalized an error-driven learning model that updates preferences to align with past choices, which makes repeating those and related choices more likely in the future. The model correctly predicts that making a free choice increases preferences along related attributes. For example, after choosing a political candidate based on trivial information (e.g., they like cats), voters' views on abortion, immigration, and trade subsequently shifted to match their chosen candidate.

## 1 Introduction

Every day, people are confronted with countless choices for which there is no objectively correct answer. These tend to be either preference judgments or moral decisions (Nakao, Ohira, & Northoff, 2012). Rather than being guided by extrinsic feedback, people choose these options freely for themselves, using their subjective preferences. We therefore refer to these choices as *free choices*. But how do people acquire these subjective preferences in the first place? The aim of this research is to understand the basis of people's subjective preferences.

We might learn about our own preferences in the same way we learn about others'; by observing and then rationalizing behaviour (Bem, 1967, Bem, 1972; Cushman, 2019). This is because we tend to lack introspective access to the mechanisms driving our behaviour, meaning that we have to post-rationalize in order to make sense of it (Bem, 1967, Bem, 1972; Mandler, 1975; Miller & Buckhout, 1973; Nisbett & Wilson, 1977). In a dramatic demonstration of this, people have been tricked by mischievous experimenters into justifying choices that they did not actually make (Hall, Johansson, Tärning, Sikström, & Deutgen, 2010; Sauerland et al., 2016; Strandberg, Sivén, Hall, Johansson, & Pärnamets, 2018). For example, after choosing their favourite flavour of jam in a taste test, participants were tricked into then justifying a different choice by experimenters, who covertly switched them mid-way through the experiment (Hall et al., 2010). Thus, rather than accessing the reasons for their choices directly, people seem to retrospectively infer them using evidence of their historic choices, even when that evidence is not valid.

As well as facilitating the inference of preferences, past choices also shape them. This has been demonstrated in studies of free choice, which show that after freely choosing an option, people tend to increase their subjective preference for it (Akaishi, Umeda, Nagase, & Sakai, 2014; Alos-Ferrer & Shi, 2012; Ariely & Norton, 2008; Brehm, 1956; Chammat et al., 2017; Cockburn, Collins, & Frank, 2014; Koster, Duzel, & Dolan, 2015; Miyagi, Miyatani, & Nakao, 2017; Nakao et al., 2016; Riefer, Prior, Blair, Pavey, & Love, 2017; Schonberg et al., 2014; Sharot, De Martino, & Dolan, 2009; Vinckier et al., 2019; Voigt, Murawski, & Bode, 2017). In the original free-choice paradigm, Brehm (1956) asked participants to rate a set of items (e.g. snack products), choose between two similarly rated options and finally to rate the full set again. Results showed that after making the forced choice, they had an increased preference for the chosen item on the final rating and a decreased preference for the rejected item. This is surprising, because it suggests that merely choosing or rejecting an option causes a person to update their subjective preference for it.

Although there has been some debate as to the validity of the free-choice paradigm in its original format, more recent studies have suggested that choice-based learning is real. For example, one particular concern about the original paradigm was that the first rating phase was noisy and therefore an imperfect measure of people's true preferences (Chen & Risen, 2010; Izuma & Murayama, 2013). However, researchers have since overcome this concern using various methodological adaptations, demonstrating choice-induced preference change does occur (Akaishi et al., 2014; Alos-Ferrer & Shi, 2012; Koster et al., 2015; Miyagi et al., 2017; Nakao et al., 2016; Schonberg et al., 2014; Sharot, Velasquez, & Dolan, 2010; Vinckier et al., 2019) and can be long-lasting (Sharot et al., 2009). For example, Sharot et al. (2010) asked participants to blindly choose between masked holiday destinations, which were only revealed to participants after one of two keys had been pressed. Subsequent ratings of those destinations were consistent with choice-induced preference change, even though choices had been randomly assigned to participants. More recent analyses of data collected from supermarket shoppers in-the-wild gives further credence to the claim that choices are self-reinforcing. In particular, a recent study of 283,000 British consumers found that their tendency to repeat a choice (i.e., *exploit*) strengthened as a function of the number of previous repetitions (Riefer et al., 2017). The consensus from studies inside and outside the laboratory is that free choices appear to be self-reinforcing, such that people come to prefer the options they choose.

**Fig. 1:**
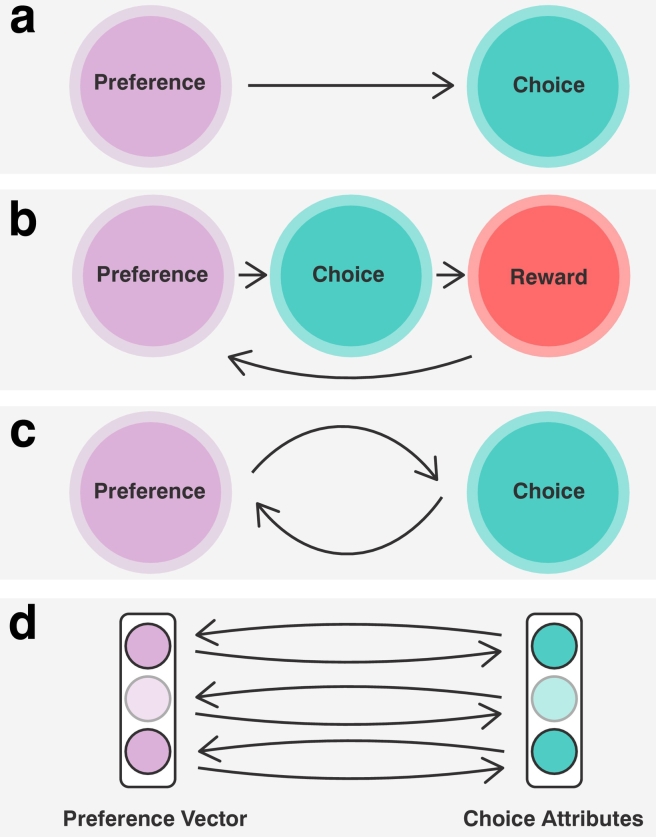
Many popular models of decision-making cannot easily explain how people form strong subjective preferences for free choices made in the wild. **a**, In standard decision theory, it is assumed that preferences remain stable over time (Glimcher, 2009; von Neumann, Morgenstern, & Rubinstein, 1944). Indeed, for many researchers, the challenge is often learning what people's preferences *are* (e.g., by asking them to choose between options), rather than understanding how they *became*. **b**, Reinforcement Learning (RL) models contrast in that they assume preferences change over time. Specifically, RL agents learn to prefer actions with a higher expected reward, which they learn as they monitor extrinsic feedback from their environment (Sutton & Barto, 1998). While RL has been shown to account for many aspects of human learning well (for a review, see Daw & Tobler, 2013), these investigations have been largely confined to objective tasks, where there is a clear extrinsic signal steering the decision-maker. **c**, Studies of free choice — where there is no objectively correct answer — have shown that merely choosing an item increases one's preference for it. This has often been taken to imply that free-choices are self-reinforcing, so as to increase preferences towards the chosen option. Yet, caching values in this way would arguably not scale well, as it would require people to keep track of every item they'd ever tried. **d**, In this paper, we propose a novel theory of subjective preference formation and decision making that arguably scales better to free choices made in the real world. According to this theory, people encode preferences over attributes of free choices, such as the hop content in beer. After making a decision, they then update their preferences in the direction of the attributes that defined their prior choice, thereby increasing their preference for it, as well as for other options that have similar attributes.

Studies of free choice imply that people refer to past acceptances and rejections to infer what they like and dislike (Akaishi et al., 2014; Chammat et al., 2017; Cockburn et al., 2014; Izuma et al., 2010; Miyagi et al., 2017; Riefer et al., 2017). Yet, we know that people can also infer the value of things they've never tried. For example, one could infer that they would not enjoy sky diving, despite having never tried it. How do people do this? Rather than caching the value of individual options (e.g. beer varieties), people likely represent options and preferences within a shared, continuous, multidimensional space (e.g., varieties of hop, brand and brewing style) (Hornsby, Evans, Riefer, Prior, & Love, 2019). As depicted in Fig. 1, representing options and preferences in this way is beneficial, in that it provides a lower-dimensional learning problem and allows one to infer the relative value of any option in their environment, irrespective of whether it has been tried.

Making free choices may therefore serve a considerably broader function than first thought, helping us to learn more deeply about ourselves and the world around us. Specifically, if options are represented within the same attribute space, then free choices may help to determine where one's preferences lie within that space. In this paper, we propose that the position of one's preferences is determined by a general, error-driven learning process, where the error term seeks to make the last choice more likely to repeat. As well as increasing the likelihood of past choices being repeated — as has been shown in real supermarket consumers (Riefer et al., 2017) — one should also increase their preferences for other options to the extent that they are *similar* to those previously chosen. While surprising, striving for internal coherency in this way may make sense in a world where choices can be evaluated across a multitude of different criteria.

We begin by demonstrating how the intrinsic desire to *maximize coherency* between past choices and present preferences can elicit strong subjective preferences in the absence of extrinsic reinforcement. In accordance with our proposed theory, we develop a computational cognitive model that learns preferences over choice attributes and uses past choices as the basis for updating them. We call it the Coherency Driven Choice (CDC) model. CDC is similar to models in the field of human category learning, which are primarily concerned with classification of items into a set of mutually exclusive categories via their attributes (e.g., using wings, beaks and feathers to describe birds) (Kruschke, 1992; Love, Medin, & Gureckis, 2004; Nosofsky, 2011). However, rather than updating based on corrective feedback, our model self-supervises using its past choices, thereby making them and similar options more likely to be sampled. Moreover, we use these mechanisms to make decisions that do not involve any fixed set of classes; the model chooses a set of items, which is not of fixed size. Through simulation, we show how this mechanism can drive complex, multidimensional preferences from free choices alone. Thus, error-driven, self-supervision helps the agent to maximize coherency between its preferences and choices over time. As a result, CDC can achieve a sense of order in environments where there are innumerable possible options and dimensions by which to score them.

After presenting this formal demonstration of our theory, we validate its predictions using a large-scale experiment of human participants. Chiefly, the error-driven nature by which CDC learns means that it will update its preferences in order to maximize the perceived contrast between accepted and rejected options. This is analogous to contrastive learning effects documented within the field of category learning, where the experience of contrasting category exemplars causes perceived category averages to drift apart and become idealized (Davis & Love, 2010). Results from Experiment 1 demonstrated that people update their preferences in a similar way following a choice. Specifically, participants were shown to prefer never-before-seen patterns if they happened to be on the back of a toy robot they had just designed. The more discriminating the pattern was to the initial robot, the more likely they were to prefer it.

Whereas Experiment 1 concerned preference formation in a novel and well-controlled domain, Experiment 2 evaluated the model's predictions in a domain in which people hold strong, preexisting preferences. In particular, Experiment 2 evaluated whether participants would retrospectively update their political beliefs following a vote. Results revealed that after choosing between two electoral candidates based on trivial grounds (e.g., whether they liked cats or dogs), participants were more likely to agree with a political belief later revealed by their chosen candidate, irrespective of whether that belief was traditionally left or right wing (e.g., pro-choice vs. pro-life abortion rights). Thus, these results support the key claims of our proposed theory, in that they suggest that people retrospectively update their preferences to be coherent with their past choices. Significantly, this even occurs in domains where people possess strong prior preferences that likely have strong subjective significance.

## 2 The coherency driven choice (CDC) model

We begin by formally describing our model of subjective preference learning and decision making.

Broadly speaking, the model works by maintaining an internal set of preferences and attention weights for attributes across choices. For example, all products in a supermarket can be described in terms of nutritional attributes such as salt, sugar and saturated fat content. Individuals will possess different preferences for those attributes, and pay differing levels of attention to them. These preferences and attention weights are used to determine how favourable a choice is at a given timepoint. In particular, the higher the attention-weighted similarity, the more likely it will be to choose that option. Our model can be thought of as an agent interacting with its environment. Much like a reinforcement learning agent, the model takes an action, observes its environment, updates its internal state and then repeats the process.

Here we introduce some important notation relevant to the model's decision making process. Note that vectors will now be denoted in bold lowercase letters and matrices in bold uppercase letters. We denote the observation of choices in the environment using the matrix *O*, which has a shape of *N* × *M*. Here, *N* denotes the number of choices available *O* = [*o*_1_, *o*_2_, …, *o*_*N*_]^*T*^ at a given timestep. For simplicity of notation, we assume that the model must choose between two items at any one time (i.e. *N*=2). However — in principle — the model is not constrained to this. *M* denotes the number of attributes for each option. Thus, each column *o*_*i*_ (*i* ∈ {1,...*N*}) is a vector of *M* attributes *o*_*i*_ = [*o*_*i*1_, *o*_*i*2_, …, *o*_*iM*_]. Therefore, the element *o*_*ij*_ corresponds to the *j*th attribute (*j* ∈ {1,...*M*}) of the *i*th item.

#### 2.1.1 Preference similarity

In order to determine the most appropriate choice, the model first calculates a probability over the available options observed in *O* using the preference vector *p* = [*p*_1_, *p*_2_, …, *p*_*M*_]^*T*^ and the attention weight vector *w* = [*w*_1_, *w*_2_, …, *w*_*M*_]^*T*^. Each element of the preference and attention weight vectors *p*_*j*_ and *w*_*j*_ maps to an attribute *j* in the attribute vector *o*_*ij*_.

After sampling an option from the environment, the agent must update the preference and attention weight vectors. We now discuss the process of computing probabilities, selecting actions and updating vectors in more detail.

#### 2.1.2 Choice probabilities

In order to determine the probability of an action, the model calculates an attention-weighted similarity between the preference vector and each of the *N* = 2 item vectors *o*_*i*_ within the observation matrix *O*. We denote the attention-weighted similarity as *a*(*o*_*i*_)

(1) aoi≡−γ∑j=1Mwjoij−pj212

where *γ* is a scaling hyperparameter. Note that this weighted Euclidean similarity term is very similar to the one used in the ALCOVE model of human category learning (Kruschke, 1992).

In order to determine the probability of selecting an option *i*, the attention-weighted similarity *a*(**o**_*i*_) is then fed into a softmax function

(2) $$f\left(o_{i}\right)\equiv \mathbb{P}\left(I_{i}|O\right)\equiv \mathrm{softmax}{\left(a\left(O\right)\right)}_{i}=\frac{\exp a\left(o_{i}\right)}{\sum_{k=1}^{N}\exp a\left(o_{k}\right)}$$

Thus, the preference *a*(*o*_*i*_) for an option is a function of three things:

1. The similarity (i.e. Euclidean distance) between the preference vector *p* and the choice attribute vector *o*_*i*_ — The more similar the attributes and corresponding preference values, the more the model prefers that option
2. The attention weight vector *w* — A higher degree of attention towards a similarity leads to a greater impact on the overall preference
3. The scaling hyperparameter *γ* — The higher the *γ*, the higher the probability for selecting the preferred option (when using softmax action selection)

Similar to more traditional models, preferences are represented as ideal-points within a multidimensional space (Greenhoff & Mac Fie, 1994). However, unlike many of those methods, the model can have varying levels of attention to those according to the attention weights and — crucially — describes how preferences update over time as a consequence of decision making.

#### 2.1.3 Action selection

Choices can be selected using one of the many popular strategies used in RL, such as *ε*-greedy, softmax action selection (Sutton & Barto, 1998) or more sophisticated directed-exploration strategies (e.g., uncertainty minimization). In each case, higher probabilities for choices (i.e. stronger preferences) increase the likelihood of exploiting that known favourite, rather than exploring disfavoured options. When using softmax selection specifically, the *λ* parameter can be thought of as determining the “fussiness” of the agent's choices, such that higher *λ* equates to a higher likelihood of choosing the favourite. We denote the choice made by the agent as *c*.

#### 2.1.4 Updating preference and attention-weight vectors

Following an action, the agent must then update its preference and attention weight vectors. As discussed in the main text, a battery of psychological research has shown that — in subjective choice domains where there is no explicit feedback — preferences tend to follow choices. We therefore update the preference and attention weight vectors so as to maximize the likelihood of the previous choice. This contrasts sharply with traditional preference models, which seldom specify how preferences may change over time (DeSarbo & Kim, 2012; Greenhoff & Mac Fie, 1994).

The exact learning procedure used to update the preference and attention weight vectors is gradient descent on the cross-entropy loss, similar to that used during backpropagation and in the neural network literature generally (Goodfellow, Bengio, & Courville, 2016; Hinton, McClelland, & Rumelhart, 1986). During the learning procedure, an action is determined probabilistically using the softmax choice rule. After the action, the cross-entropy loss is calculated between the preference probabilities output by the model **f**(**o**_*i*_) and the actual choice *c* that was made.

(3) $$l\left(f\left(O\right),c\right)\equiv -\sum_{i=1}^{N}1_{\left\{c=i\right\}}\;\log\left(f\left(o_{i}\right)\right)$$

After making an action, the preference and attention weights are updated so as to minimize the cross-entropy error. Concretely, they are updated proportionally to the negative of the error gradient.

We therefore use the following calculation to find the partial derivative of the preference vector **p** with respect to the cross-entropy loss:

(4) $$\frac{\partial l\left(f\left(O\right),c\right)}{\partial p_{j}}\equiv \gamma^{2}w_{j}\sum_{i=1}^{N}\left(1_{\left\{c=i\right\}}-f\left(o_{i}\right)\right)\frac{1}{a\left(o_{i}\right)}\left(o_{\mathrm{ij}}-p_{j}\right)$$

And the following calculation to find the partial derivative of the attention weight vector *w* with respect to the cross-entropy loss:

(5) $$\frac{\partial l\left(f\left(O\right),c\right)}{\partial w_{j}}\equiv -\frac{\gamma^{2}}{2}\sum_{i=1}^{N}\left(1_{\left\{c=i\right\}}-f\left(o_{i}\right)\right)\frac{1}{a\left(o_{i}\right)}{\left(o_{\mathrm{ij}}-p_{j}\right)}^{2}$$

We then use these partial derivatives to update the existing preference and attention weight vectors using gradient descent. Concretely, we define the following update rules for the vectors **p** and **w**, respectively:

(6) $$p≔p-\eta_{p}\frac{\partial l\left(f\left(O\right),c\right)}{\partial p_{j}}$$

(7) $$w≔w-\eta_{w}\frac{\partial l\left(f\left(O\right),c\right)}{\partial w_{j}}$$

where *η*_*p*_ and *η*_*w*_ represent the learning rates for the preference and attention weight vectors, respectively. As is standard during gradient descent, these learning rates scale the updated vectors and thus determine the magnitude of the update at a given time step.

## 3 Simulating free choices demonstrates how strong preferences can be learned over time

To illustrate how one could learn strong subjective preferences by virtue of their choice trajectory, we simulated the CDC model. In the simulated environment, there were two choice types that did not vary on the first dimension but varied significantly on the second dimension. This is analogous to choosing between two beer brands that are similar in taste but contrast in the colour of branding.

### 3.1 Method

#### 3.1.1 Simulation

Observations in the environment were randomly sampled from distributions of two clusters. Choice *type a* had a cluster centroid of (0.2,0.8) while *choice type b* had a cluster centroid of (0.2,0.2). The standard deviation of each cluster was determined apriori to be 0.05; thereby making the two choice types linearly separable. A total of 500 observations were simulated.

The agent was initialised with middling preferences and attention weights across the two attributes of (0.5,0.5). It was also set to have a learning rate of 0.01, *ε*=0.05 and *λ*=1. Choices were simulated for 10,000 timesteps. At each timestep, the agent was forced to choose between two randomly-selected options from each choice type using an *ε*-greedy action selection strategy.

Choosing these parameters over a large number of timesteps helped the learning process to be smooth and stable across the agent's lifespan. This is therefore more representative of what might happen over a timescale of several years. However, it should be noted that the same learning trajectory documented below could be found using a larger learning rate over a shorter number of timesteps, though many such situations would not involve complete movement towards a goal.

### 3.2 Results and discussion

**Fig. 2:**
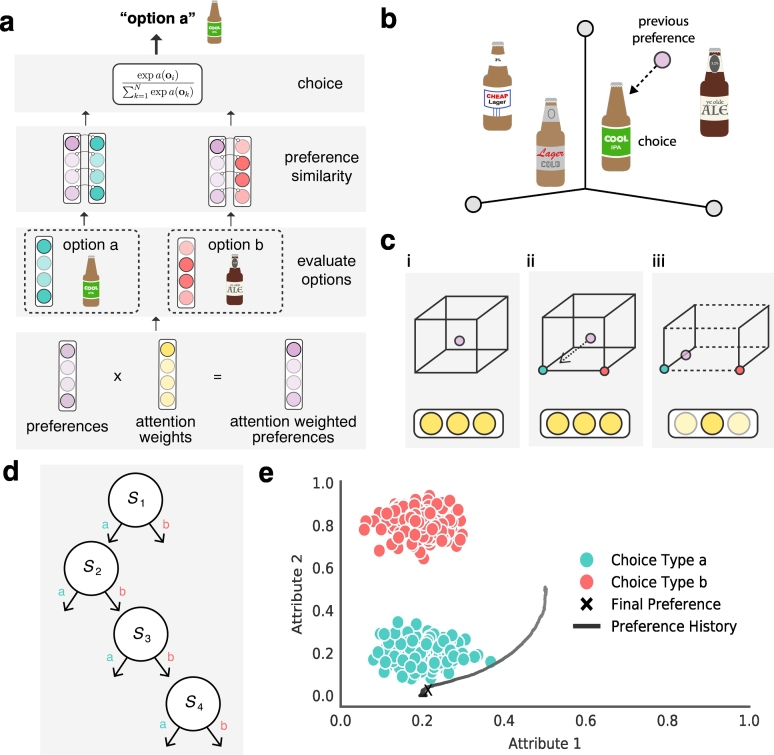
To illustrate how this theory can materialize in strong subjective preferences, we formalize it in a computational cognitive model, known as Coherency Driven Choice (CDC). **a**, CDC possesses a preference and attention-weight vector. When evaluating options, the model evaluates the similarity between its own attention-weighted preference and the attributes of the available options. The closer an option's attributes are to the model's attention-weighted preferences, the more likely the model is to select it. **b**, Following a choice, CDC updates its preference vector to make the past choice most likely using gradient descent, thereby moving towards the prior choice in attribute space. **c**, CDC also adjusts its attention weights to make prior choice more likely, effectively warping the preference space so that it becomes more sensitive to the attended attribute. **d**, As the model grows to prefer the options it initially chose, it tends to repeat the same choice type more over time. **e**, The model was simulated for 10,000 timesteps in a simple environment in which there were two choice types. Due to happenstance in the initial choices, the model began to prefer *choice type a*, adjusting its preferences and attention weights in favour of the attributes that make it unique. The preference history is coloured by attention weight ratio, such that blacker colors indicate a greater deal of attention paid towards attribute 2.

A visualization of this model and the results of this simulation are shown in Fig. 2.

After simulating 10,000 forced-choices, CDC eventually came to posses preferences resembling the first choice type (i.e., *type a*). After an initial sequence of random actions, CDC began to quickly develop a preference in retrospect of them, and thus select choices consistent with this new-found preference. This process was then self-reinforcing, further strengthening preferences and therefore the likelihood for choice *type a* over time. Thus, when consumers become less likely to explore new products the more they exploit the same, it may be because their preferences are being self-reinforced by their past choices, as shown in this simulation (Riefer et al., 2017).

Uniquely, as CDC chose more of *choice type a*, the preferences and attention of the model moved most in favour of the attributes of the choice that made it unique. This is a known consequence of discrimination learning, but uniquely demonstrated here within the context of subjective preference change (Davis & Love, 2010; Ramscar, Yarlett, Dye, Denny, & Thorpe, 2010; Rescorla & Wagner, 1972). Exaggerating preferences in this way helped the model to maximize the perceived contrast between the accepted and rejected choices. This increases the likelihood of the past choice type being sampled again and reduces the likelihood of the rejected option being selected. Continuing the example introduced above, this suggests that a person will have an over-exaggerated preference towards the unique branding of their preferred beer, helping to retrospectively justify their apparent preference.

Of course, when formalizing a cognitive theory, one must make some assumptions about the world. For example, in the case presented here, one could argue that people do not always have complete knowledge of the attributes describing each option at the time of decision. Indeed, one may need to taste a product to know how salty it is, or they may vote for a political candidate before learning of their stance on free-trade. In this case, our model would simply use a placeholder for their preference on that particular attribute. Upon revelation of the attribute value for their prior choice (e.g., discovering how salty a snack was), they would then move their preference towards the attribute point in question.

In reality, people are unlikely to develop preferences as exaggerated as the ones learned in this simulation. This is because choices in real life are often more innumerate, multidimensional and overlapping. The high degree of similarity between options in the real world would cause our agent to explore more, and thereby develop a less extreme set of preferences. The environment may also provide additional sources of noise during decision making that elicit exploration and thus movement of preferences in new directions. For example, a preferred product may be out-of-stock in a store, a person may develop a new allergy or travel to a new country. Rather than attempt to account for the multitude of ways in which preferences change as a function of choices in-the-wild, the aim of this simulation was to highlight a important consequence of the coherency maximizing mechanism proposed here; namely, that preferences are updated in order to discriminate the choice just made.

## 4 People prefer novel patterns associated with their prior choice

The new account proposed in this paper suggests that by learning preferences over attributes of choices, people can generalize their preferences to novel options that are associated with ones previously chosen. Moreover, similar to error-driven models developed in the field of category learning (Davis & Love, 2010), it predicts that people will update their preferences in the direction of the most discriminative elements of their choice, in order to maximize the likelihood it being repeated.

**Fig. 3:**
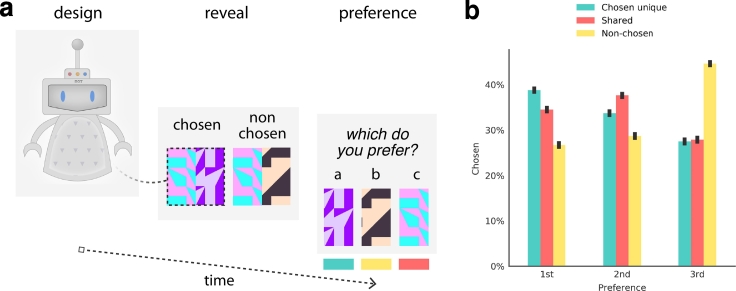
**a**, The trial sequence observed by participants. First, participants were introduced to a robot and asked to design aspects of it. They were then introduced to a new robot, which was designed differently. These robots then turned around, revealing random patterns on their backs. One pattern was unique to the robot participants' had previously designed (i.e., chosen-unique), one was shared across both robots (i.e., shared) and the other was unique to the robot they had previously eschewed (i.e., non-chosen). Participants were then asked to rank the patterns in order of preference. **b**, This chart depicts the proportion that each image type was chosen as a first, second and third preference (with standard error bars). These results are consistent with the theory presented here, which predicts that choice-based learning generalizes most strongly to the unique, most-discriminating feature of the original choice (i.e., chosen unique), followed by the shared feature.

Experiment 1 aimed to validate these predictions. Here, participants were asked to design a robot (the trial flow is depicted in Fig. 3a). They were then introduced to a second robot, before both turned around revealing previously unseen, randomly assigned patterns on their backs. Finally, participants were asked to choose between three patterns; one that was unique to the back of the robot they had previously designed (i.e. chosen unique), another that was shared across the backs of the two robots and a final pattern that was unique to the back of the robot they had not designed (i.e. non-chosen). It was hypothesized that — consistent with a discriminative account of learning — participants would prefer those novel patterns to the extent that they were uniquely associated with the robot they had just designed.

### 4.1 Method

#### 4.1.1 Participants

One thousand and three participants were recruited from Amazon Mechanical Turk (mturk). Mturk (www.mturk.com) is generally known for being an inexpensive source of reliable human data (Crump, McDonnell, & Gureckis, 2013, though see McDuffie (2019) for a discussion on the possible limitations). Participants were required to have completed >1000 tasks (or *HITs*) and have an acceptance rate >95%. Participants had to be based in the US or Canada. Data from 37 participants were removed due to having an average response time two standard deviations greater than the mean (> 17.64 s). The mean age of the participants was 37.0 (*SD* = 11.4) and 50.2% were male. Participants were paid 50 for participating, which is typical for mturk (Horton & Chilton, 2010). Overall, the experiment took about 10 min.^1^

#### 4.1.2 Design

The experiment used a between-groups design with 10 trials. Participants either chose a pattern that belonged to the back of the robot they previously designed (i.e., *chosen-unique*), the one they did not design (i.e., *non-chosen*) or was shared across both. The dependent variable was therefore the sum of the preferences over each of the choice types across each of the 10 trials.

#### 4.1.3 Apparatus & stimuli

The study was designed using JavaScript and was accessed in a web browser. The task was presented in a 700 × 700 pixel screen. During the design phase of the experiment, participants responded by choosing attributes from a drop-down box. When stating their design preferences in the final phase, participants were asked to click each robot in order of their preference (from highest to lowest).

Each robot was designed using the Support Vector Graphics (SVG) format. These robots had a front — which could be designed by participants — and a back, which contained randomly-assigned patterns.

During the design phase, participants could design three aspects of the front of their robot; the stomach texture, the visor border colour and the eye colour. In each trial, participants could choose between two randomly-selected options for each design aspect.

In the final phase of each trial, participants were shown multicoloured geometric patterns. In total, there were three patterns per trial, randomly selected from one of 50 possible triplets. One pattern appeared on one random half of the back of the robot they had previously designed. Another pattern appeared on one random half of the back of the robot they had not designed. The final design was shared such that it appeared on both remaining halves of the two turned robots.

#### 4.1.4 Procedure

Participants were initially briefed about the experiment in order to get their informed consent and asked to supply their age and gender. They were told that the task would take about 10 min and would require them to design robots and make choices. On each trial, participants were shown a robot with a randomly selected name and asked to design it. The hope was that by designing the robot, they would become more motivated about their choice, increasing ecological validity. They could choose between one of two randomly selected options for each of the three design aspects. After completing the design, participants were then introduced to a new, frowning *anti-robot*. This anti-robot was designed using all the attributes that the participant had previously eschewed. In addition, participants were warned that the anti-robot did not like the participants' design. They were then asked to reassure their own robot by clicking on it. Henceforth, we refer to this anti-robot as the *non-chosen* option. This is because it was uniquely designed using elements that had been explicitly rejected during the previous phase of the trial. After clicking on their own robot, both robots then turned around revealing randomized patterns on their backs (described above). The two robots then moved to the back of the screen. Three patterns — either shared across both or unique to one of the robots at the back of the screen — then appeared at the front of the screen and participants were asked to choose their favourite in order of preference. Participants completed 10 such trials with patterns, robot names, and other trial details randomized for each trial, they were debriefed, thanked for their participation and paid immediately.

### 4.2 Results and discussion

A repository for all data described in this paper is available on OSF ([dataset] Hornsby & Love, 2019).

The proportion of times each image type was selected as a first, second and third preference is pictured in Fig. 3b. As hypothesized, participants most preferred the unique chosen pattern, followed by the shared pattern and finally, the non-chosen pattern (omnibus non-parametric Friedman test of differences among repeated-measures *χ*^2^=137.48,*p*<0.001). Specifically, summed preferences for the chosen-unique patterns (Median = 9, *IQR* = 4.0) were stronger than that for the shared items (*Median* = 9, *IQR* = 3.0) (Wilcoxon signed-rank *Z* = − 2.91, *p*<0.005,*r*=0.09) and the non-chosen items (Median = 11, *IQR* = 5.0) (*Z* = − 12.08, *p*<0.001,*r*=0.39). A final test also revealed that preferences for the shared items were stronger than that for non-chosen items (*Z* = − 11.70, *p*<0.001,*r*=0.38).^2^

These results supported our two key hypotheses and therefore the key claims of our theory. Firstly, participants exhibited an increased preference for the chosen-unique and shared patterns, demonstrating that they generalized their preference learned from the initial choice to the novel patterns by virtue of their association. Consistent with the behaviour of our model formalization, people appear to generalize their preferences to novel items that share attributes with choices just made.

Secondly, participants demonstrated an increased preference for the chosen-unique pattern over the shared pattern and a reduced preference for the pattern unique to the rejected option. This is consistent with discriminative accounts of learning, in that it suggests that people update their preference towards attributes that discriminate their prior choices. Studies of human categorization have shown that experiencing contrasting category exemplars causes their perceived difference to drift apart and become idealized. For example, because people are used to contrasting diet foods with high calorie foods, they are more likely to suggest celery as a prototypical diet food, even though it is extreme for its category (Davis & Love, 2010). Choosing freely between options appears to have similar effects during preference learning, in that preferences update to maximize the perceived contrast between the accepted and rejected options. This functions to maintain coherence between past choices and present preferences.

## 5 People adjust their existing political beliefs to be consistent with their prior vote

The experimental results presented so far have provided controlled, experimental support for our error-driven account of subjective preference formation and decision making. Outside the lab however, people usually have strong prior preferences for options, which likely interacts with their intrinsic tendency to coherency maximize.

**Fig. 4:**
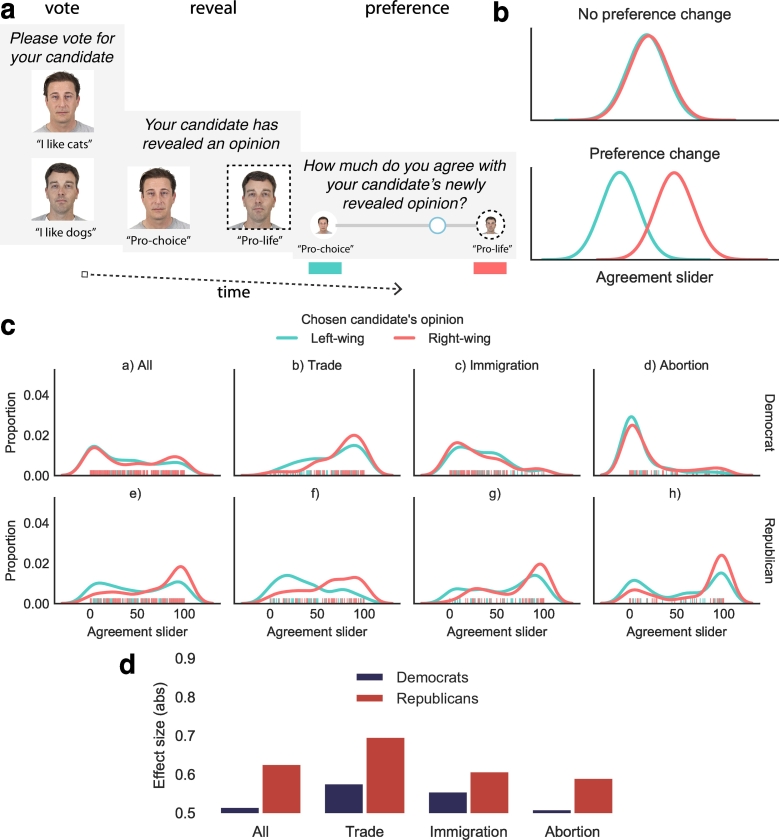
**a**, Participants were initially asked to vote for a candidate based on trivial characteristics. Both candidates then revealed opposing beliefs on a more controversial belief. Participants were then asked to rate how much they agreed with their candidate's newly revealed opinion. **b**, If voting for the candidate affects the extent to which participants agree with the later-revealed political belief, then one would expect participants to show different levels of agreement between groups, depending on whether their candidate revealed a traditionally left-wing view or a right-wing view. **c**, The results of the experiment are shown in kernel density plots (with rugplots below to depict the individual data points). These reveal that Republican-identifying participants were particularly prone to adjusting their preference to be in accordance with that revealed by their chosen candidate. **d**, Plotting effect sizes for each per-topic comparison shows that Republican-identifying participants were particularly prone to adjusting their preferences.

The aim of Experiment 2 was therefore to explore the extent to which people retrospectively update their existing preferences following a free choice. Specifically, we evaluated whether people would modify their political beliefs after a vote. Participants from the U.S. were shown two political candidates and asked to vote for one, based on some trivial attributes (e.g., whether they liked cats or dogs).^3^ This experimental procedure is depicted in Fig. 4a. Following the vote, these chosen and non-chosen political candidates revealed randomly assigned, opposing controversial beliefs on a particular topic. These beliefs were either traditionally left or right-wing. For example, if randomly assigned to the abortion issue, the chosen candidate would either show “Abortion: Pro-choice” or “Abortion: Pro-life”. Participants were then asked to state the extent to which they agreed with their chosen candidate's newly revealed belief using a slider. Finally, they were asked to state their preferred party out of the Democrats or Republicans.

Our primary hypothesis was that people would show higher levels of agreement for the right-wing view (e.g., pro-choice abortion rights) if their chosen candidate revealed support for that view, compared to the left-wing view. This would provide support for the theory presented here and suggest that, following a vote, people are prone to adjust their political beliefs to be coherent with their chosen candidate.

While expecting that people would feel inclined to agree with their chosen candidate's newly revealed opinion, we also secondarily hypothesized that this would vary depending on individual differences. Specifically, we expected that different individuals would be more or less prone to updating their preferences retrospectively, due to differences in their longstanding political beliefs. Due to its simplicity, we used people's preferred political party affiliation as an indirect measure of these beliefs and subsequently predicted that Republican-identifying participants would be more likely to update their beliefs to be coherent with their initial vote. This was for two main reasons. Firstly, due to the broad and unpredictable nature of being in power, voters in support most likely have to accept more compromises and adjust their beliefs on occasion in order to remain coherent. Secondly, results from self-reported surveys suggest that conservatives value coherency comparatively more when making political decisions (e.g. they have been shown to value loyalty to the in-group and to authority comparatively more Mendez, 2017; Haidt & Graham, 2007; Jost, Glaser, Kruglanski, & Sulloway, 2003). While this is a somewhat secondary prediction that does not directly flow from our core theory, we used Experiment 2 as an initial exploration of group differences across domains.

### 5.1 Method

#### 5.1.1 Participants

One thousand participants were recruited using mturk. All participants were required to have had at least 1000 of their previous tasks accepted on mturk, have a 95% mturk task acceptance rate and be based in the United States. All participants were paid 50 for participating. Of the participants, 47 were removed due to a lack of apparent concentration or understanding of the task. Specifically, 45 participants were removed for having response times more than two standard deviations from the mean for the vote response (*n*=26, >38.68*s*) and the slider response (*n*=19, >55.38*s*). A further 2 participants were removed for clicking >50 times during the whole experiment. Of the final 953 participants, 50.26% were male, 49.53% were female and the remainder specified as ‘other’. The mean age was 37.44 (*SD* = 11.67). Of the participants, 64.53% said they affiliated more closely with Democrats, whereas the remainder said they affiliate more closely with Republicans. Data was collected in late July 2018.

#### 5.1.2 Design

The experiment used a 2 × 2 between-groups design. The experiment involved one trial. Participants either chose a candidate that — previously unbeknownst to them — revealed a more left-wing view or a more right-wing view. Furthermore, participants either affiliated more closely with Democrats or Republicans. The dependent variable was the slider value of the participant, normalized by political direction. Specifically, these values ranged from 1 to 100, where higher values indicated more agreement with the right-wing view and lower values indicated more agreement with the more left-wing view. Note that the initial vertical location of the candidates, the allocation of neutral and controversial attributes, and the final horizontal locations of the candidates were all fully randomized.

#### 5.1.3 Apparatus & stimuli

The study was designed using JavaScript and was accessed in a web browser. The task was presented in a 1000 pixel wide screen. Participants were shown two of a possible four faces taken from the Chicago Face Database (Ma, Correll, & Wittenbrink, 2015).^4^ To control for noise emerging from other, well-documented biases, all faces were controlled to be male, Caucasian and ranging between the ages of 37 and 43. Faces were randomly assigned one of four names. These were either James Smith, Michael Johnson, John Williams, Graham Brown. These names were sourced from a list of the most popular first and second Caucasian names in the United States.

The neutral opinions of participants are shown in Table 1. The controversial political opinions of participants are shown in Table 2.

**Table 1:** A summary of the neutral statements shown to participants.

Topic	Statements
Pets	“I like cats”
	“I like dogs”
Sport	“I'm a baseball fan”
	“I'm a basketball fan”
Food	“My favourite Italian food is pizza”
	“My favourite Italian food is spaghetti”

**Table 2:** A summary of the controversial statements revealed by candidates following a vote.

Topic	Left-wing	Right-wing
Abortion	“Abortion: Pro-choice”	“Abortion: Pro-life”
Immigration	“I support policies that would increase immigration”	“I support policies that would decrease immigration”
Trade	“I support tariffs on imports”	“I support free trade”

#### 5.1.4 Procedure

Participants were initially briefed about the purpose of the experiment in order to get their informed consent. They were told that the task would take about 5 min to complete and would require them to vote for political candidates. After the briefing, participants began the trial. To first ensure that the participants were alert, they were told to read a short experimental briefing. Within this briefing was the instruction to click on the name of the university (which was displayed at the top of the screen). After clicking this, a new panel was revealed displaying some text (“It's time to vote for a candidate! Please click on a candidate to vote”) and two political candidates in gray cards; one above the other. Within each card and underneath the candidate's photographs were their name and then a list of “My opinions”. These lists initially displayed opposing neutral opinions selected from the same neutral topic, as shown in Table 1. The assignments of photographs, names, neutral topics and vertical alignment were all randomized. Participants were asked to vote for a candidate using this neutral information by clicking on their card. After voting, the cards then moved to a left or right position on the screen, aligning horizontally with each other. This horizontal allocation was also randomized. The voted and non-voted candidates then immediately revealed opposing political opinions from the same topic in their list of opinions. A blue “New” button drew attention to this newly-revealed belief. The window then moved down to reveal a new section, containing some text and a slider. The text informed participants in bold lettering that “Your candidate has revealed a new opinion above! How much do you agree with your candidate's newly revealed opinion?” Participants were prompted to use the new slider to state the extent to which they agreed with the candidates' newly revealed opinions. Small avatars of the candidate's faces were shown on either side of the slider, with a gray tick or white cross below the ones that were previously chosen, respectively. There were also prompts below each avatar reminding participants of the newly revealed controversial opinion of the candidate. Note that the slider was not initialized with any starting value. Sliding closer towards the chosen candidates indicated higher levels of agreement with that candidate. The slider had 100 possible positions. The photographs, names, neutral and political opinions and left/right direction of movement were all randomly assigned between participants.

After confirming the slider position, participants were asked to state which party they most affiliated with (Democrat or Republican), their gender and their age. Following task completion, they were thanked for their participation and paid immediately.

### 5.2 Results and discussion

In support of the primary hypotheses, results revealed that participants' stated degree of belief was significantly influenced by the randomly-assigned belief revealed by their chosen candidate (non-parametric two-way analysis of variance (ANOVA) (F (1, 952) = 28.89, *p* < 0.001, CL = 0.563). In particular, those that voted for a candidate that later revealed a right-wing opinion (Median = 56.0, IQR = 76.00) agreed an average of 43.59% more with the right-wing view compared to if the candidate later revealed a left-wing view (Median = 39.0, IQR = 69.75). This suggests that choosing a political candidate based on initially trivial characteristics made participants more likely to agree with that candidate's later-revealed controversial opinion, irrespective of whether the person self-identified as a Democrat or Republican.

In support of the secondary hypothesis, there was also a significant interaction between party affiliation and the degree of agreement with the right-wing view (*F*(1, 952) = 11.77, *p* < 0.001). A first Mann-Whitney *U* test looking at the responses of self-identifying Democrats revealed that they only slightly increased their level of agreement for the right-wing view if their candidate revealed a right-wing view (*Median* = 40.0, *IQR* = 78.00) compared to if it revealed a left-wing view (*Median* = 31.5, *IQR* = 65.75); this difference was not significant (*U* = 43,584.5, *p* = 0.054), and had a very low effect size (*CL* = 0.518). In contrast, the second test looking at Republican-identifying participants revealed a larger effect, in that — if one's candidate later revealed a more right-wing opinion — they were 65.66% more in agreement with the the right-wing view (*Median* = 82.0, *IQR* = 59.75) compared to when the candidate revealed a more left-wing view (*Median* = 49.50, *IQR* = 70.25) (*U* = 10,131.5, *p* < 0.001, *CL* = 0.629).^5^ Thus, Republican-identifying participants appeared to be particularly prone to adjusting their beliefs so as to be consistent with their last choice, even when this choice was based on trivial grounds (e.g., the fact that their candidate liked cats).

It is notable that self-identified Republicans were particularly willing to adjust their preferences to be coherent with their prior choices. For example, their median support for pro-life abortion policies was 60% higher when their chosen candidate later expressed support for pro-life policies (*Median* = 96.0, *IQR* = 61.50) compared to pro-choice policies (*Median* = 60.0, *IQR* = 88.00) (the remaining within-topic comparisons are depicted in Fig. 4c and 4d, and described in the supplemental). One possibility is that only Republican-identifying participants adjust their preferences to be consistent with a choice, across all domains. To confirm that this was not the case, we replicated the first experiment, asking about party affiliation at the end of the study. Results revealed that both self-identifying Democrat and Republican participants exhibited preferences for the final pattern in ways consistent with the reported findings above (see the supplemental for details). Thus, a more likely explanation of our results is that there are group and domain differences guiding the extent to which people learn from their past choices. For example — as discussed in the introduction to this section — differences in people's belief systems may give rise to different propensities to adjust preferences following a choice. Clearly these topics are deserving of future investigation in light of the results presented here.

## 6 General discussion

In this article, we proposed a novel account of subjective preference formation and decision making. In our model, choice options and preferences are represented in a common continuous, multi-dimensional space. When people choose options within this space, their preferences are updated to increase the likelihood of their previous choices. The objective of decision making is therefore to reduce the error between one's past choices and present preferences; we refer to this general mechanism as *coherency maximization*. Consistent with patterns of repeat-purchasing observed in supermarket consumers (Riefer et al., 2017) and studies of choice-induced preference change (Akaishi et al., 2014; Alos-Ferrer & Shi, 2012; Ariely & Norton, 2008; Brehm, 1956; Chammat et al., 2017; Cockburn et al., 2014; Koster et al., 2015; Miyagi et al., 2017; Nakao et al., 2016; Schonberg et al., 2014; Sharot et al., 2009; Vinckier et al., 2019; Voigt et al., 2017), coherency maximization boosts the likelihood of past choices being repeated by shifting preferences towards the chosen item and away from rejected alternatives. As was shown in a simple simulation, this mechanism can drive strong subjective preferences in the absence of extrinsic feedback. Thus, our model is well-suited to navigating the complex choices encountered in everyday life.

Following from this account, one would expect preferences to be exaggerated towards attributes that favoured the past choice and diminished towards attributes associated with rejected choices. Results from the first experiment showed that preferences are updated in accordance with these predictions. Concretely, the more uniquely associated a novel pattern was to the back of a toy robot they had previously designed, the more likely participants were to prefer it. Given that we tend to lack direct introspective access to the mechanisms driving our behaviour, post-rationalizing choices may be a reasonable way to make sense of ourselves and the world around us (Bem, 1967, Bem, 1972; Cushman, 2019; Mandler, 1975; Miller & Buckhout, 1973; Nisbett & Wilson, 1977). Our model shows that simple learning processes can achieve similar ends by updating underlying preferences to align with a choice.

These learning rules can be seen as reflecting an internal drive for internal consistency, which is pivotal to rational models of decision making (Savage, 1954). For example, it is often assumed that subjective choices should be stochastically transitive (Schultz, Dayan, & Montague, 1997). When preferences are not transitive, people can become liable to manipulation (or “dutch booking”) (Davidson, McKinsey, & Suppes, 1955). For example, if out of three beers, a person prefers beer A over B, beer B over C but would rather have C than A, their preferences are cyclical. This person could be tricked into paying for a series of costly trades in which the drinker ended with his original beer. In subjective domains, internal consistency may be at times the only rational strategy that is feasible.

The second experiment explored how the tendency to maximize coherency interacted with people's prior preferences. The results indicated that after voting for a political candidate based on trivial criteria (e.g., the candidate likes cats), participants were more likely to agree with a controversial opinion later revealed by the candidate, such as their stance on abortion rights. This held up as a main effect, supporting our claim that people retrospectively update their preferences over attributes of their past choices to make them more likely. The fact that this was particularly pronounced for Republican-identifying participants supported our secondary hypothesis that this mechanism can vary between groups, consistent with previous studies demonstrating individual differences in choice-induced preference change (Cockburn et al., 2014). However, while individual differences may partly explain our results, it is likely that other variables outside the scope of our theory also influence decisions, such as how important affiliation is to different groups. Specifically, while Republican-identifying participants adjusted their preferences more than their Democrat-identifying counterparts in the second experiment, a replication of the first experiment (see Supplement) revealed no difference between the groups when choosing between novel options. In future research, the prominence of choice-induced preference change within certain groups or domains could be estimated by fitting the CDC model to different decision tasks.

If people update their preferences to be coherent with their past choices, then why might they also feel motivated to explore? A recent analysis of consumers' take-away purchases suggested that they were more likely to try a different restaurant after a positive experience rather than repeat it again (Schulz et al., 2019), suggesting that people may also explore to reduce uncertainty about their environment. In the simulation, we used a stochastic, undirected exploration policy (i.e. *e*-greedy). However, CDC could be adapted to use a more sophisticated, directed exploration strategy, such as uncertainty minimization. This is because CDC considers exploration and coherency maximization as theoretically and mechanistically independent. Understanding where and when people explore is an open question in the literature (for a review, see Hills et al., 2015), meaning that such adaptations would need to be evaluated with scrutiny. In the future, we hope to further understand how people trade-off this need to explore with the desire to coherency maximize.

In this article, we have assumed that preferences are adjusted following a choice. However, preference change has also been shown to occur “online” during a choice (Akaishi, Kolling, Brown, & Rushworth, 2016; Niv et al., 2015; Schonberg et al., 2014; Voigt, Murawski, Speer, & Bode, 2019). A recent study by (Voigt et al., 2019) found that choice-induced preference changes only occurred for choices that were remembered. Both online and post-choice induced preference change mechanisms could co-exist. Though this would highlight a future area of development for the CDC model. One such modification would be to adapt the role of the attention weights during a choice. For example, their influence could be magnified in cases where the model was more familiar with or remembered the attributes of the choice. The preference vectors and attention weights would then account for both online and post-decisional effects of preference change, respectively.

Although our studies involved brief decision-making episodes, the basic mechanisms considered here may also apply at longer timescales. Indeed, this work was partially motivated by Riefer et al.'s (Riefer et al., 2017) discovery of self-reinforcing purchasing patterns in supermarket consumers, which extended over several months. If CDC's predictions for how preferences change held over extended periods of time, the practical consequences and possibilities for behavioural change would be substantial.

For instance, CDC predicts that purchasing the same *type* of food should increase preference for associated attributes. This can be problematic in cases where the food is unhealthy (e.g., high in sodium or saturated fat). For example, studies of nutrition have shown that repeated exposure to a particular ingredient (e.g., sodium) increases one's desire and lowers their sensitivity to it, making it difficult for them to adjust when they go on a diet (Bertino, Beauchamp, & Engelman, 1982; Mattes, 1993; Liem & de Graaf, 2004). The advent of targeted recommender systems means that these cyclical effects may be being perpetuated further. While recommender systems often reduce diversity of alternatives in the environment (Pariser, 2011), coherency maximization causes preferences to become less diverse, thereby perpetuating the problem. Rather than blindly targeting people based on their previous consumption, marketers could incorporate external objectives to their targeting algorithms. For example, our theory predicts that recommending healthy alternatives that are similar to people's existing preferences could lead to long-term improvements in their choices.

The modeling approach presented here is readily extended to account for the richness of people's preferences. Unlike how CDC was formalized here, people are unlikely to have a single preference across each respective attribute. For example, while a foodie might prefer expensive, locally produced foods, they may also be happy to watch affordable, mass produced television. People may have different preferences for the same attribute (e.g., cost) depending on context. Fortunately, it would be straightforward to extend CDC to have multiple preference vectors to capture this context dependence, similar to how models of human category learning possess multiple clusters in which only the most contextually relevant one is updated during a learning episode (Love et al., 2004). Such a model would cast coherency maximization as a process that occurs within a domain (e.g., food, entertainment, etc.) as opposed to globally.

The model presented here may also be extended to account for some well-known decision biases. For example, CDC predicts that people will prefer novel options when they are similar to options that have repeatedly tried in the past. Thus, it may be able to account for the mere-exposure effect, where people have been shown to prefer options by virtue of their familiarity (Zajonc, 1968). Similarly — though only distantly related to the free choices described here — it is possible that CDC could be adapted to account for anchoring effects. For example, forcing CDC to update its preferences towards a given anchor would cause it to become more favourable towards related options (see attitudinal change accounts of anchoring for related arguments, e.g., Wegener, Petty, Detweiler-Bedell, & Jarvis, 2001). While such relationships are speculative at this stage, they indicate a general, coherency-driven learning mechanism may underpin several, well-known sequential choice biases.

In conclusion, preferences and choices can be characterized as existing within a common space. While we prefer options that match our preferences, we also appear to engage in error-driven learning to update our preferences to accommodate our past choices. Because preference and choice representation lie in a shared multidimensional space, the choices we make have consequences (i.e., spillover effects) for related future choices. For example, people may be prone to agree with a controversial opinion held by a political candidate, by virtue of the fact that they voted for them. Although this behaviour may appear irrational, being internally coherent may be the best we can hope for in complex, subjective domains. Being aware of these coherency maximizing dynamics may make it possible for people to ameliorate some of the potentially harmful consequences. For example, if a voter chooses a candidate based on tax policy, perhaps being mindful of that fact will make the voter less likely to reflexively adopt their candidate's positions on unrelated issues. Likewise, these same coherency maximizing principles could be incorporated into recommender systems to help consumers achieve some positive goal, such as eating more healthily.

## CRediT authorship contribution statement

**Adam N. Hornsby:** Conceptualization, Methodology, Software, Formal analysis, Investigation, Writing - original draft, Visualization, Writing - review & editing, Resources, Validation. **Bradley C. Love:** Conceptualization, Writing - review & editing, Supervision, Project administration, Funding acquisition.

## Appendix A Supplementary data

Supplementary material Image 1

## Footnotes

1 Both experiments presented here were in compliance with UCL's code of ethics

2 All Wilcoxon-signed rank tests were evaluated against a Holm-Bonferroni corrected alpha value for multiple comparisons

3 More information about the experimental design and stimuli is available in the methods section

4 These faces can be identified in the Chicago Face Database using the target codes WM-023, WM-221, WM-223 and WM-248

5 For all multiple comparisons reported in this paper, significance values were compared against a Holm-Bonferroni corrected *α* value
