# Supplementary material for: How decisions and the desire for coherency shape subjective preferences over time
Source: Cognition. 2020 Jul;200:104244. doi: 10.1016/j.cognition.2020.104244 (PMC7315129; doi:10.1016/j.cognition.2020.104244)
Supplement: Supplementary file 1 — Supplementary material [file mmc1.pdf]

Supplementary Information

Experiment 1: People prefer novel patterns associated with their prior choice

Preferences did not change over time. One possible confound in the robot experiment is that participants became more likely to select a particular image as their first preference over trials. In order to evaluate this claim, preferences for each choice type were broken down by trial. Three post-hoc linear regressions conducted on each choice type showed non-significant effects of trial when predicting the number of selections ($p > 0.05$), suggesting that participants did not become biased in their choices over time.

Preference change doesn't vary as a function of political affiliation. One possible explanation for the results discovered in experiment two (i.e., the study of political opinions) is that only self-identifying Republicans adjust their preferences to be coherent with their past choices. To test this claim, the first study was re-run, this time asking participants to state their preferred political party of the Democrats and the Republicans at the end of the experiment.

The re-run used the same participant selection criteria as the first iteration, except that participants were required to be from the United States only. Of the remaining 953 participants, 54.77% were female. The mean age of participants was 38.80 (SD = 12.18). This time, the experiment was conducted in May 2019.

Firstly, results showed a direct replication of the overall effect. The initial omnibus test proved significant (non-parametric Friedman test of differences among repeated-measures $\chi^2 = 104.75, p < 0.0001$). As before, summed preferences for the chosen-unique patterns (Median = 9, $IQR = 4.0$) were stronger than that for the shared items ($Median = 10$, IQR = 3.0) ($Z = -4.10, p < 0.0001, r = 0.133$) and the non-chosen items (Median = 11, IQR = 5.0) ($Z = -10.99, p < 0.0001, r = 0.36$). A final test also revealed that preferences for the shared items were stronger than that for non-chosen items ($Z = -9.77, p < 0.001, r = 0.32$)[1].

Looking at self-identifying Democrat participants alone ($N = 625$), results showed the same pattern of results as described above, suggesting that they update their preferences to be in line with their prior choices (non-parametric Friedman test of differences among repeated-measures $\chi^2 = 81.05, p < 0.0001$). As before, summed preferences for the chosen-unique patterns (Median = 9, $IQR = 4.0$) were stronger than that for the shared items ($Median = 10$, $IQR = 3.0$) ($Z = -3.42, p = 0.0006, r = 0.14$) and the non-chosen items (Median = 11, $IQR = 5.0$) ($Z = -9.40, p < 0.0001, r = 0.376$). A final test also revealed that preferences for the shared items were stronger than that for non-chosen items ($Z = -8.36, p < 0.0001, r = 0.33$).

[1] All Wilcoxon-signed rank tests were evaluated against a Holm-Bonferroni corrected alpha value for multiple comparisons

Similarly for Republican-identifying participants alone ($N = 328$), results showed the same pattern of results as described above (non-parametric Friedman test of differences among repeated-measures $\chi^2 = 25.45, p < 0.0001$). As before, summed preferences for the chosen-unique patterns (Median = 9, $IQR = 4.0$) were stronger than that for the shared items ($Median = 10$, $IQR = 3.25$) ($Z = -2.25, p = 0.0245, r = 0.12$) and the non-chosen items (Median = 11, $IQR = 5.0$) ($Z = -5.74, p < 0.0001, r = 0.32$). A final test also revealed that preferences for the shared items were stronger than that for non-chosen items ($Z = -5.11, p < 0.0001, r = 0.28$).

Thus, there is no evidence to suggest that only self-identifying Republicans update their preferences to be maximally coherent with their past choices.

Experiment 2: People adjust their existing political beliefs to be consistent with their prior vote

Preference change varies within political topic. Further experimental results from the study of people's political preferences are now reported. First, additional results from the main ANOVA are reported. Then, analyses are broken down by political topic.

As the slider response is an ordinal variable, the data was analyzed using a non-parametric two-way analysis of variance (ANOVA) (Hocking, 1985) [2]. This ANOVA compared the influence of two between-groups independent variables (selected candidate opinion and political affiliation) on the participants' normalized slider values. Political affiliation contained two levels (Democrat or Republican identifying), as did the selected candidate opinion (left-wing or right-wing).

In addition to the results presented in the main text, the ANOVA revealed a main effect of political affiliation. This yielded an F-ratio of $F(1, 952) = 83.72$, p < 0.001, $CL = 0.640$[3], indicating that the average slider values of self-identifying Democrats (*Median* = 34.0, *IQR* = 73.00) were significantly lower than that of Republicans (*Median* = 67.0, *IQR* = 70.75).

Going further, we now evaluate how peoples levels of agreement varied depending on both their party affiliation and the topic that participants were being asked to decide on. Breaking down by individual topic allows us to gain further insight about the malleability of certain beliefs held by self-identifying Democrats and Republicans. Therefore, for these groups separately, a further three Mann-Whitney U tests were run, comparing the extent to which the normalized slider values changed depending on the revelation of left and right-wing opinions in each of the three topics.

[2] We are grateful to the creators of the *RFit* package for implementing this for the *R* programming language

[3] For all main effects and Mann-Whitney U tests, we report the common language (*CL*) effect size (Mcgraw & C. P. Wong, 1992)

For Democrat-identifying participants, participants only appeared to be affected by the revelation of their chosen candidate's beliefs if it pertained to trade or abortion. Results showed that normalized slider values of self-identifying Democrats were significantly lower on average if their candidate later revealed a left-wing opinion about trade (*Median*=73.0, *IQR* = 47.00) compared to a right-wing opinion about trade (*Median*=82.0, *IQR*=29.00) (U = 4688.5, $p<0.01$, *CL* = 0.579). Similarly, results showed that responses of Democrat-identifying participants were significantly different if their candidate later revealed a left-wing opinion about abortion (*Median* = 1.0, *IQR*=13.00) compared to a right-wing opinion about abortion (*Median* = 3.0, *IQR*=27.50) (U = 4311.5, $p<0.02$, *CL* = 0.512). However, responses of Democrat-identifying participants were not significantly different if their candidate later revealed a left-wing opinion about immigration (*Median*=29.5, *IQR*=38.25) compared to a right-wing opinion about immigration (*Median*=24.0, *IQR*=44.25) (U = 4352.5, $p>0.05$, *CL* = 0.442).

For Republican participants, the average normalized slider values were significantly different across all topics depending on the revealed opinion of the chosen candidate. Results showed that normalized slider values of Republicans were significantly lower on average if their candidate later revealed a left-wing opinion about trade (*Median*=31.0, *IQR* = 43.5) compared to a right-wing opinion about trade (*Median*=67.0, *IQR*=51.50) (U = 1023.5, $p<0.001$), *CL* = 0.699). In addition, results showed that responses of Republican participants were significantly lower if their candidate later revealed a left-wing opinion about abortion (*Median* = 60.0, *IQR*=88.00) compared to right-wing opinion about abortion (*Median* = 96.0, *IQR*=61.5) (U = 1136.0, $p<0.01$, *CL* = 0.593). Finally, responses of Republican participants were significantly lower on average if their candidate later revealed a left-wing opinion about immigration (*Median*=65.5, *IQR*=61.25) compared to a right-wing opinion about immigration (*Median*=84.5, *IQR*=55.5) (U = 1080.0, $p<0.015$, *CL* = 0.610).

Responses were not biased in favour of a particular party affiliation or slider response. Post-hoc analyses were conducted to test whether there were any biases in responses or whether the neutral topics elicited any differences in slider responses. A 2x2 chi-square was conducted to ensure that the process of voting did not alter participant's political affiliation. Results showed that voting for a candidate that later revealed a left or right-wing opinion did not affect participants' subsequent self-reported party affiliation ($X^2(1)$=1.583, $p>0.05$). In addition, a one-sample Wilcoxon signed-rank test was conducted to ensure that participants did not have a bias towards responding in any particular direction (e.g. clicking more towards the left). Results revealed that participants' average, unnormalized slider values (*Median* = 50, *IQR* = 76.00) were not significantly different from 50 (W = 218707.5, $p > 0.05$).

References

Hocking, R. R. (1985). *The analysis of linear models.* Brooks/Cole.

Mcgraw, K., & C. P. Wong, S. (1992, 03). A common language effect size measure. *Psychological Bulletin*, *111*, 361-365. doi: 10.1037/0033-2909.111.2.361
